# Infection with *Batrachochytrium dendrobatidis* is common in tropical lowland habitats: Implications for amphibian conservation

**DOI:** 10.1002/ece3.5098

**Published:** 2019-04-01

**Authors:** Héctor Zumbado‐Ulate, Adrián García‐Rodríguez, Vance T. Vredenburg, Catherine Searle

**Affiliations:** ^1^ Department of Biological Sciences Purdue University West Lafayette Indiana; ^2^ Departamento de Zoología, Instituto de Biología Universidad Nacional Autónoma de México (UNAM) Ciudad de México México; ^3^ Escuela de Biología Universidad de Costa Rica San José Costa Rica; ^4^ Departamento de Ecologia Universidade Federal do Rio Grande do Norte Natal Brazil; ^5^ Department of Biology San Francisco State University San Francisco California

**Keywords:** amphibians, *Batrachochytrium dendrobatidis*, Chytridiomycosis, conservation, lowlands, population declines

## Abstract

Numerous species of amphibians declined in Central America during the 1980s and 1990s. These declines mostly affected highland stream amphibians and have been primarily linked to chytridiomycosis, a deadly disease caused by the chytrid fungus *Batrachochytrium dendrobatidis* (Bd). Since then, the majority of field studies on Bd in the Tropics have been conducted in midland and highland environments (>800 m) mainly because the environmental conditions of mountain ranges match the range of ideal abiotic conditions for Bd in the laboratory. This unbalanced sampling has led researchers to largely overlook host–pathogen dynamics in lowlands, where other amphibian species declined during the same period. We conducted a survey testing for Bd in 47 species (*n* = 348) in four lowland sites in Costa Rica to identify local host–pathogen dynamics and to describe the abiotic environment of these sites. We detected Bd in three sampling sites and 70% of the surveyed species. We found evidence that lowland study sites exhibit enzootic dynamics with low infection intensity and moderate to high prevalence (55% overall prevalence). Additionally, we found evidence that every study site represents an independent climatic zone, where local climatic differences may explain variations in Bd disease dynamics. We recommend more detection surveys across lowlands and other sites that have been historically considered unsuitable for Bd occurrence. These data can be used to identify sites for potential disease outbreaks and amphibian rediscoveries.

## 1 INTRODUCTION

Globally, biodiversity is decreasing at an alarming rate even in seemingly pristine and protected environments (Barnosky et al., 2011; Novacek & Cleland, 2001). Species declines are driven by numerous anthropogenic actions, acting alone or synergistically with natural threats (Hooper et al., 2012; Rödder, Kielgast, & Lötters, 2010; Sala et al.., 2000). Previous studies suggest that immediate conservation efforts should prioritize actions on endangered taxa that are rapidly declining and the habitats that protect these species (Brooks et al., 2006; Foden et al., 2013; Giraudo & Arzamendia, 2018). However, there is often incomplete information on which populations are suffering the greatest declines and which locations provide them with the best chances of long‐term persistence. For example, for several endangered species or clades, the majority of conservation actions have been designed based on opportunistic field studies conducted in sites where historic declines occurred (Kriger & Hero, 2007a). The potential bias caused by this unbalanced sampling might lead researchers to overestimate the rate of decline or to miss less dramatic declines and environmental threats across the range of the declining species. Therefore, extending the sampling to heterogeneous habitats across the entire geographic distribution of threatened species is crucial to detect and quantify potential threats as well as to establish suitable and more effective conservation actions (Hitchman, Mather, Smith, & Fencl, 2018; Miller et al., 2018; Olson et al., 2013).

Historic research on global amphibian population declines provides numerous examples of conservation actions in response to environmental threats in specific ecosystems. During the last four decades, at least 43% of described amphibian species declined or became extinct worldwide from multiple causes (Collins, 2010; Monastersky, 2014; Stuart et al., 2004; Wake & Vredenburg, 2008; Young et al., 2001). One widespread cause of amphibian population declines is the introduction of infectious pathogens. For example, *Batrachochytrium dendrobatidis* (Longcore, Pessier, & Nichols, 1999) (hereafter Bd) is a fungus that causes chytridiomycosis, a deadly cutaneous disease that affects amphibians in all continents where amphibians occur (Berger et al., 1998; Fisher, Garner, & Walker, 2009). Global assessments conservatively estimate that chytridiomycosis has caused the severe decline or extinction of over 200 species (Skerratt et al., 2007). Highland stream‐dwelling amphibians have been hypothesized to be more prone to massive Bd‐related die‐offs than amphibians in other habitats (Hero, Williams, & Magnusson, 2005; Hirschfeld et al., 2016; Lips, 1998; Lips, Reeve, & Witters, 2003). Evidence suggests that tropical highland stream environments match the range of ideal abiotic conditions where Bd reproduces best in the laboratory (Berger et al., 2004; Longcore et al., 1999; Piotrowski, Annis, & Longcore, 2004). However, the spatial dynamics of Bd are intricate and still poorly understood. It is known that the intensity and occurrence of epizootic outbreaks and length of negative effects upon amphibian communities have varied globally (Catenazzi, 2015). In addition, numerous field studies show that prevalence and intensity of Bd infection vary with host species, microhabitat, temperature, humidity, seasonality, and geographic location (Kinney, Heemeyer, Pessier, & Lannoo, 2011; Kriger & Hero, 2007b; Kriger, Pereoglou, & Hero, 2007; Phillott et al., 2013; Searle, Gervasi et al., 2011). Thus, identifying conditions that constrain the geographic distribution of this pathogen will help elucidate why some species and populations suffer declines from Bd and identify locations that may be environmental refuges from infection (Murray et al., 2011; Rödder, Veith, & Lötters, 2008; Rosenblum et al., 2013).

**Figure 1:**
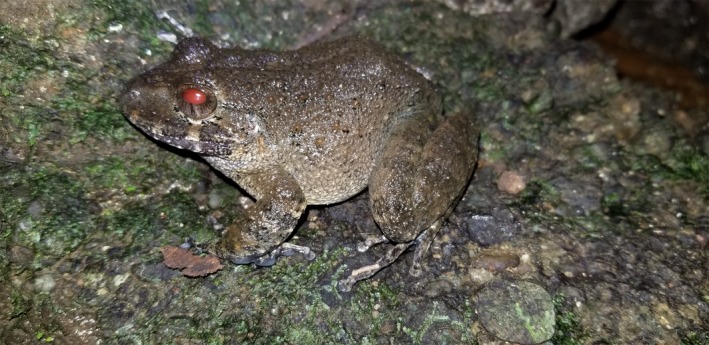
Female individual of the Critically Endangered Golfito robber frog (*Craugastor taurus*). This species was very common in lowlands of Southern Costa Rica but catastrophically declined during the 1980s and 1990, presumably due to chytridiomycosis. Currently, it is only present in Punta Banco (one of our study sites) and Puerto Armuelles (Panama)

The strong elevational gradients in the mountain ranges of Central America (Savage, 2002) create habitat heterogeneity and high endemism of amphibians in midlands and highlands (>800 m elevation). The cool and moist environments in tropical highlands provide suitable conditions for the Bd epizootic that occurred in Central America during the 1980s and 1990s, causing the extinction of an unknown number of amphibian species, especially highland stream‐breeding species (Cheng, Rovito, Wake, & Vredenburg, 2011; Lips, Diffendorfer, Mendelson, & Sears, 2008; Pounds et al., 2006; Pounds & Crump, 1994; Rovito, Parra‐Olea, Vasquez‐Almazan, Papenfuss, & Wake, 2009). Historical declines in montane amphibian species reflect why most studies on amphibian host‐Bd dynamics in the tropics have been conducted in premontane and upper elevation localities (Lips, 1999,1998; Puschendorf, Bolaños, & Chaves, 2006; Ryan, Lips, & Eichholz, 2008). For example, a considerable amount of Bd infection data has been opportunistically collected from montane ecosystems, increasing the focus of conservation actions on highlands while overlooking other potential environments where amphibians may also be impacted by Bd (Puschendorf, Hodgson, Alford, Skerratt, & VanDerWal, 2013). For example, the suitability of lowland ecosystems for the spread of Bd has been frequently disregarded (Puschendorf et al., 2009) even though is known that some amphibian species (Figure 1) and clades have suffered dramatic unexplained declines in these zones (Chaves et al., 2014; La Marca et al., 2005; Puschendorf et al., 2009; Ryan et al., 2008; Whitfield et al., 2007; Zumbado‐Ulate, Bolaños, Gutiérrez‐Espeleta, & Puschendorf, 2014).

Despite the focus on highlands for most Bd‐related studies, the few studies conducted in lowlands of Central America have found new locations where this pathogen occurs, suggesting that Bd is more widely distributed than previously thought (Flechas, Vredenburg, & Amézquita, 2015; Kilburn et al., 2010; von May, Catenazzi, Santa‐Cruz, & Vredenburg, 2018; Whitfield et al., 2013; Whitfield, Kerby, Gentry, & Donnelly, 2012; Woodhams et al., 2008; Zumbado‐Ulate et al., 2014). Predictive models and abiotic suitability for Bd across heterogenous landscapes (Brannelly, Martin, Llewelyn, Skerratt, & Berger, 2018; García‐Rodríguez, Chaves, Benavides‐Varela, & Puschendorf, 2012; Puschendorf et al., 2009; Rödder et al., 2008) can be generated using available bioclimatic databases such as WorldClim. This dataset contains 19 bioclimatic variables generated by land area interpolations of climate point data from 1950 to 2000. These variables were derived from monthly precipitation and temperature data at weather stations around the world and describe annual means (e.g., annual precipitation and temperature) and average of extreme environmental values (e.g., maximum temperature of warmest month) (Hijmans, Cameron, Parra, Jones, & Jarvis, 2005). Thus, combining information on infection prevalence and abiotic conditions (e.g., from the WorldClim dataset) across the entire geographic distribution of a host can provide a more informative distribution of both the host and pathogen to identify potential hotspots of future disease outbreaks and potential environmental refuges from disease (Green, 2017; James et al., 2015; Rödder et al., 2010).

In this study, we sampled for Bd at four tropical lowland locations in Costa Rica and contrasted Bd prevalence and intensity of infection across study sites. We hypothesized that different host–pathogen dynamics occur across study sites because they exhibit latitudinal and altitudinal variation (Kriger & Hero, 2008; Kriger et al., 2007). We extracted all 19 bioclimatic variables of the WorldClim to describe the different ranges of temperature and precipitation across study sites, which are the main environmental variables that affect Bd growth and dispersal (Nowakowski et al., 2016; Savage, Zamudio, & Sredl, 2011). Additionally, we hypothesized that all study sites would exhibit low levels of Bd prevalence and intensity of infection suggesting stable enzootic infections of Bd (Retallick, McCallum, & Speare, 2004; Scheele, Hunter, Brannelly, Skerratt, & Driscoll, 2017; Woodhams et al., 2008). Finally, we also expected a higher prevalence of Bd in amphibian assemblages occurring in permanent streams than in ephemeral ponds and terrestrial assemblages, as has been found in previous studies (Kriger & Hero, 2007a; Lips et al., 2003).

## 2 METHODS

### 2.1 Lowland sampling sites

**Figure 2:**
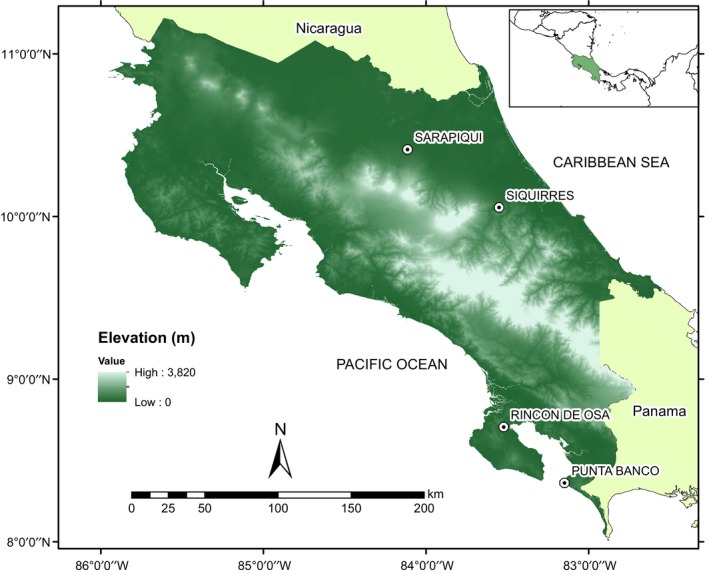
Map of Costa Rica showing elevational gradient and lowland sites surveyed for *Batrachochytrium dendrobatidis*

We sampled four assemblages of amphibians between November and December 2011, at four tropical lowland locations in Costa Rica (Figure 2). We defined tropical lowlands as all tropical locations within 0–800 m elevation according the Holdridge Life Zone System (Holdridge, 1967). Study sites consisted mostly of tropical moist forest and tropical wet forest with transitional ecosystems including semi‐deciduous and evergreen forests, with temperature and precipitation ranges characteristic of these life zones. Our four sampling sites grouped into two main zones:

#### 2.1.1 Caribbean zone

Here, we sampled at Tirimbina Private National Wildlife Refuge at La Virgen, Sarapiqui, on the north Caribbean lowlands (10.41N, −84.11W, 0–200 m elevation), and at the Costa Rican Amphibian Research Center, at Guayacan, Siquirres (10.06N, −83.55W, 400–600 m elevation).

#### 2.1.2 Pacific zone

Here, we focused on the areas surrounding the small towns of Rincon de Osa (8.71N, −83.52W, 0–50 m elevation) and Punta Banco (8.36N, −83.15W, 0–50 m elevation), where we sampled across patches of coastal forest. Our sampling in this zone was limited because we were only able to access private farms upon the authorization of landowners.

### 2.2 Pathogen detection

At each site, four people systematically searched for amphibians for 36–48 hr during the day and night (9–12 hr/person). Within each site, we conducted visual encounter surveys of amphibians (Heyer, Donnelly, McDiarmid, Hayek, & Foster, 1994) and classified them by the habitat where they were captured: stream‐dwellers (permanent flowing water), pond‐dwellers (standing ephemeral waterbodies such as swamps, pools, and ditches), and forest‐dwellers (leaf‐litter, tree holes, or bromeliad plants in the understory, and canopy). Caught amphibians were stored individually in clean, unused plastic bags. Each individual was inspected for visible signs of chytridiomycosis, such as hyperplasia, hyperkeratosis, abnormal shedding, depigmentation, and lethargic behavior (Berger et al., 1998; Voyles et al., 2009) and swabbed to detect Bd with a cotton swab (Medical Wire and Equipment, MW–113) using nitrile gloves. To swab, we ran a total of 20 strokes on every individual as follows: five strokes on one hand, five strokes on the ventral patch, five strokes on one foot, and five strokes along inner thigh. Swabs were stored dry in 1.5 ml Eppendorf tubes and frozen at −20°C until DNA extraction. All amphibians were immediately released after sampling. During this study, we followed field protocols (Kriger, Hines, Hyatt, Boyle, & Hero, 2006; Skerratt et al., 2008) which were approved by the National System of Conservation Areas of Costa Rica (SINAC, research permit 001–2012–SINAC) which ensures that animals are being cared for in accordance with standard protocols and treated in an ethical manner.

We extracted DNA from swabs using PrepMan Ultra (Boyle, Boyle, Olsen, Morgan, & Hyatt, 2004). All extractions were diluted 1:10 in 0.25X TE buffer and run in singlicate (Kriger, Hero, & Ashton, 2006) following diagnostic quantitative PCR (qPCR) standard protocols (Boyle et al., 2004) using an Applied BioSystems Prism 7300 Sequence Detection System to test for the presence and quantity of Bd genome equivalents. All Bd‐positive samples were run again in singlicate confirmatory assay. Negative controls (DNase/RNase‐free distilled water) were run in triplicate on every 96‐well PCR plate. We used 100, 10, 1, and 0.1 zoospore quantification standards to produce a quantification curve. We multiplied the qPCR score by 80 to calculate the zoospore genomic equivalents in the original sample and calculated the average value from the two singlicate assays (Vredenburg, Knapp, Tunstall, & Briggs, 2010; Warne, LaBumbard, LaGrange, Vredenburg, & Catenazzi, 2016).

### 2.3 Data analysis

We were interested in understanding how Bd prevalence and intensity varied among our study sites and habitats (predictor variables). For our analyses, we pooled all species together instead of using species as predictor or running independent tests for each species because the samples sizes per species were highly variable (from 1–44). This high variance in the sample size could produce significant models that may be an artifact of opportunistic sampling instead of a real pattern. Therefore, we analyzed habitat as a proxy of amphibian community composition, because the species variable was 100% correlated to habitat. To contrast Bd prevalence, we used fix‐effects generalized linear models (GLMs) to find the most suitable model using binomial response variables (infected or not infected). Candidate models were ranked according to the Akaike's information criterion (AIC) to determine the relative importance of predictor variables within each model set. The model with the lowest AIC was considered the most robust (Burnham & Anderson, 2004). To compare infection intensity among locations and habitats (predictors), we generated fix‐effects general linear models (LMs) with data only from infected individuals. We built our models using the log‐transformed Bd load (estimated number of genomic equivalents) as a response variable and included site and habitat as predictors. Candidate models were ranked according to the coefficient of regression (*R* ^2^), with the model with the highest R^2^ considered the most robust (Zar, 2013). For the most robust GLM, we tested the significance of the predictors using an ANOVA with a chi‐square approximation to find the probabilities of predictor variables within the most suitable models, and for the most robust LM we used an ANOVA. Finally, we conducted post hoc, pairwise comparisons (Tukey test) to confirm where the differences occurred between significant predictors.

To describe the local abiotic environment for the sampled lowland sites, we generated buffers (radius = 10km) around each one of our four study sites. Because we wanted to achieve a full description of the abiotic environment, we extracted values for all the cells occurring within each buffer (mean = 355 cells/site, Table 1) from all 19 bioclimatic variables of WorldClim (version 1.4; www.worldclim.org) at a spatial resolution of 30 arc‐s (Hijmans et al., 2005). We compared the abiotic environment among sites using a principal component analysis (PCA). To contrast climatic dissimilarities between lowland study sites, we also generated a pairwise matrix of Euclidean distances between the centroids of climatic envelopes. All analyses were conducted in R v.3.5.1 (R Core Team, 2014).

**Table 1:** Mean values (standard deviation) of the 19 bioclimatic variables from the WorldClim dataset and loads (coordinates) for PCA axes 1 and 2 showing the specific contribution of each of the bioclimatic variables used in the environmental analysis of four lowland sites in Costa Rica

Bioclimatic variables	Punta Banco	Rincon de Osa	Sarapiqui	Siquirres	PC1	PC2
BIO_1_ = Annual Mean Temperature	25.5 (0.7)	25.6 (0.6)	25.4 (0.7)	24.4 (1.1)	0.1	−0.1
BIO_2_ = Mean Diurnal Range	10.1 (0.7)	11.0 (0.2)	9.0 (0.0)	9.0 (0.0)	0.0	−0.4
BIO_3_ = Isothermality	75.4 (0.9)	76.6 (0.7)	77.3 (0.7)	79.4 (0.7)	−0.1	0.4
BIO_4_ = Temperature Seasonality	77.9 (5.5)	78.0 (1.8)	73.3 (5.6)	76.1 (2.5)	0.0	−0.6
BIO_5_ = Max Temperature of Warmest Month	32.8 (0.8)	33.2 (0.7)	31.6 (0.7)	30.4 (1.1)	0.1	−0.5
BIO_6_ = Min Temperature of Coldest Month	19.2 (0.9)	18.9 (0.9)	19.8 (0.7)	19.0 (1.2)	0.1	0.1
BIO_7_ = Temperature Annual Range	13.8 (1.0)	14.2 (0.4)	12.0 (0.2)	11.2 (0.4)	0.0	−0.6
BIO_8_ = Mean Temperature of Wettest Quarter	25.0 (0.7)	25.1 (0.7)	25.3 (0.9)	24.2 (1.2)	0.1	−0.1
BIO_9_ = Mean Temperature of Driest Quarter	25.8 (0.6)	25.8 (0.7)	25.9 (0.7)	25.1 (1.2)	0.1	−0.1
BIO_10_ = Mean Temperature of Warmest Quarter	26.6 (0.8)	26.7 (0.8)	26.4 (0.8)	25.5 (1.1)	0.1	−0.2
BIO_11_ = Mean Temperature of Coldest Quarter	24.7 (0.8)	24.8 (0.8)	24.6 (0.6)	23.6 (1.1)	0.1	−0.1
BIO_12_ = Annual Precipitation^a^	3,112.0 (134.0)	3,976.4 (430.3)	4,085.4 (185.5)	3,784.4 (245.8)	128.1	31.4
BIO_13_ = Precipitation of Wettest Month^a^	586.3 (47.2)	712.7 (51.4)	460.4 (19.1)	440.1 (23.5)	13.8	−49.9
BIO_14_ = Precipitation of Driest Month	54.0 (14.8)	60.7 (19.5)	163.6 (13.4)	182.1 (18.7)	3.6	24.8
BIO_15_ = Precipitation Seasonality	64.5 (6.4)	62.8 (4.9)	30.0 (1.5)	27.2 (2.1)	−0.7	−7.4
BIO_16_ = Precipitation of Wettest Quarter^a^	1,351.0 (87.7)	1,719.3 (130.8)	1,277.3 (56.9)	1,173.9 (65.2)	41.7	−88.5
BIO_17_ = Precipitation of Driest Quarter^a^	176.8 (49.1)	237.4 (65.2)	589.9 (40.9)	625.1 (53.4)	14.8	82.8
BIO_18_ = Precipitation of Warmest Quarter	528.5 (27.6)	707.5 (82.4)	724.5 (41.9)	772.7 (72.4)	21.7	19.6
BIO_19_ = Precipitation of Coldest Quarter^a^	1,071.8 (132.0)	1,348.7 (152.2)	1,163.9 (71.9)	1,089.1 (60.4)	38.4	−36.0

Notes

Temperature variables are measured in Celsius (environmental variables 1–11) and precipitation variables in mm (environmental variables 12–19).

a Bioclimatic variables with higher contribution.

## 3 RESULTS

We screened a total of 348 adult amphibians from 47 species for Bd (346 frogs and two salamanders, Table 2). From this list, a total of 44 species are classified as least concern and three are categorized as threatened: *Oophaga granulifera* is classified as vulnerable (VU), *Agalychnis lemur* and *Craugastor taurus* are classified as critically endangered (CR) according the International Union for Conservation of Nature (IUCN) (Red List of Threatened Species, version 2017–1; http://www.iucnredlist.org/). Overall, 33 species (70.2% of sampled species) tested positive for Bd and total prevalence of Bd was 54.6%. We did not detect Bd on three of the amphibian families sampled, including Plethodontidae, the only family of Salamanders in the Neotropics; however, the sample size for these families was very small.

**Table 2:** List of species and number of individuals tested for *Batrachochytrium dendrobatidis* in amphibian assemblages from four lowland sites in Costa Rica

Species	Habitat	*N* (Bd positive)	Prevalence % (95% CI)	Genomic equivalents (±*SE*)		
				Sarapiqui	Siquirres	Punta Banco
*Agalychnis callidryas*	Pond	11 (5)	45.5 (16.7–76.6)	x	249.2 ± 214.1	x
*Agalychnis lemur* a	Pond	5 (2)	40.0 (5.3–85.3)	x	12.3 ± 4.9	x
*Agalychnis spurrelli*	Pond	5 (1)	20.0 (5.0–71.6)	x	10.3 ± 0.0	x
*Anotheca spinosa*	Forest	1 (1)	100.0 (0.2–100.0)	x	112.3 ± 0.0	x
*Boana rufitela*	Pond	10 (8)	80.0 (44.4–97.5)	8.4 ± 3.9	x	x
*Bolitoglossa colonnea*	Forest	1 (0)	0.0 (0.0–97.5)	x	x	x
*Centrolenella ilex*	Stream	1 (1)	100.0 (0.2–10.00)	x	57.4 ± 0.0	x
*Cochranella granulosa*	Stream	1 (1)	100.0 (0.2–100.0)	3.9 ± 0.0	x	x
*Craugastor bransfordi*	Forest	24 (19)	79.2 (57.8–92.9)	31.6 ± 13.8	74.9 ± 112.2	x
*Craugastor crassidigitus*	Forest	6 (2)	33.3 (4.3–77.7)	3.0 ± 0.0	18.5 ± 0.0	
*Craugastor fitzingeri*	Forest	44 (26)	59.1 (43.2–73.7)	448.8 ± 321.2	14.1 ± 5.6	65.4 ± 22.5
*Craugastor megacephalus*	Forest	2 (1)	50.0 (12.6–98.7)	0.6 ± 0.0	x	x
*Craugastor mimus*	Forest	10 (8)	80.0 (44.4–97.5)	107.5 ± 76.9	x	x
*Craugastor stejnegerianus*	Forest	6 (2)	33.3 (4.3–77.7)	x	x	2.2 ± 0.9
*Craugastor taurus* a,b	Stream	15 (12)	80.0 (51.9–95.7)	x	x	11,632.5 ± 6,285.2
*Cruziohyla calcarifer*	Forest	1 (0)	0.0 (0.0–97.5)	x	x	x
*Dendrobates auratus*	Forest	7 (1)	14.3 (0.4–57.9)	x	4.9 ± 0.0	x
*Dendropsophus ebraccatus*	Pond	22 (15)	68.2 (45.1–86.1)	x	130.3 ± 59.1	x
*Dendropsophus phlebodes*	Pond	1 (1)	100.0 (0.2–10.0)	x	15.9 ± 0.0	x
*Diasporus diastema*	Forest	9 (4)	44.4 (13.7–78.8)	x	1994.3 ± 1724.7	x
*Diasporus vocator*	Forest	1 (0)	0.0 (0.0–97.5)	x	x	x
*Duellmanohyla rufioculis*	Stream	1 (0)	0.0 (0.0–97.5)	x	x	x
*Engystomops pustulosus*	Pond	10 (0)	0.0 (0.0–30.8)	x	x	x
*Hyalinobatrachium fleischmanni*	Stream	1 (0)	0.0 (0.0–97.5)	x	x	x
*Hyalinobatrachium valerioi*	Stream	1 (0)	0.0 (0.0–97.5)	x	x	x
*Hyloscirtus palmeri*	Stream	1 (1)	100.0 (0.2–100.0)	x	231.2 ± 0.0	x
*Incilius melanochlorus*	Pond	8 (1)	12.5 (0.3–52.6)	3.3 ± 0.0	x	x
*Leptodactylus fragilis*	Pond	1 (0)	0.0 (0.0–97.5)	x	x	x
*Leptodactylus insularum*	Pond	3 (0)	0.0 (0.0–70.7)	x	x	x
*Leptodactylus poecilochilus*	Pond	1 (0)	0.0 (0.0–97.5)	x	x	x
*Leptodactylus savagei*	Pond	3 (0)	0.0 (0.0–70.7)	x	x	x
*Lithobates vaillanti*	Pond	2 (0)	0.0 (0.0–84.2)	x	x	x
*Lithobates warszewitschii*	Stream	26 (14)	53.8 (33.4–73.3)	51.8 ± 39.1	1,391.1 ± 704.7	x
*Oedipina gracilis*	Forest	1 (0)	0.0 (0.0–97.5)	x	x	x
*Oophaga granulifera* a	Forest	1 (1)	100.0 (0.2–100.0)	x	x	114.0 ± 0.0
*Oophaga pumilio*	Forest	23 (18)	78.3 (56.3–92.5)	625.2 ± 479.5	x	x
*Pristimantis cerasinus*	Forest	7 (4)	57.1 (18.4–90.1)	3.6 ± 0.5	x	x
*Pristimantis ridens*	Forest	6 (3)	50.0 (11.8–88.2)	3.0 ± 0.0	6.4 ± 3.2	x
*Rhaebo haematiticus*	Stream	27 (17)	63.0 (42.4–80.6)	3.1 ± 0.8	x	x
*Rhinella horribilis*	Pond	4 (0)	0.0 (0.0–60.2)	x	x	x
*Scinax boulengeri*	Pond	4 (1)	25.0 (63.1–80.6)	195.2 ± 0.0	x	x
*Scinax elaeochroa*	Pond	6 (3)	50.0 (11.8–88.2)	x	2.3 ± 0.4	x
*Smilisca phaeota*	Pond	5 (2)	40.0 (5.3–85.3)	x	9.8 ± 2.3	x
*Smilisca sordida*	Stream	1 (1)	100.0 (0.2–100.0)	430.4 ± 0.0	x	x
*Tlalocohyla loquax*	Pond	15 (11)	73.3 (44.9–92.2)	x	1,566.8 ± 1,020.7	x
*Teratohyla spinosa*	Stream	4 (2)	50.0 (6.8–93.2)	4.8 ± 1.2	x	x
*Teratohyla pulverata*	Stream	3 (1)	33.3 (84.0–90.6)	x	39.8 ± 0.0	x
Total		348 (190)	54.6 (49.2–59.9)			

Notes

For every species, the table shows the habitat where the species was captured, the sample size, the overall prevalence (95% CI), and the average (*SE*) of genomic equivalents of *Batrachochytrium dendrobatidis* zoospores quantified per study site estimated from Bd‐positive samples.

a Endangered species according the International Union for Conservation of Nature (IUCN).

b Prevalence value previously reported in Chaves et al. (2014).

**Figure 3:**
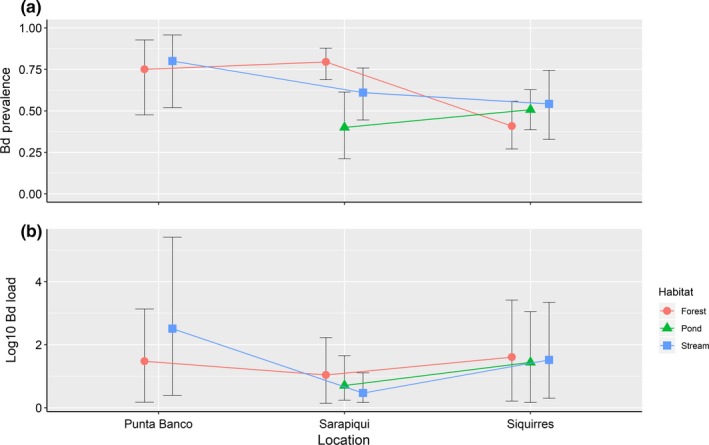
Prevalence and intensity of infection of *Batrachochytrium dendrobatidis* in amphibian assemblages from four surveyed lowland sites in Costa Rica. The line plots show (a) prevalence of *B. dendrobatidis* among surveyed lowland sites per habitat (with 95% binomial confidence intervals) and (b) average infection intensity (*SE*) of *B. Dendrobatidis* in amphibian assemblages among surveyed lowland sites per habitat. The figure does not show results for Rincon de Osa because Bd prevalence at that site was 0%. Similarly, the plots do not display results for the category pond at Punta Banco because we did not collect any individuals from ponds at that location

Prevalence of infection showed high heterogeneity among sites with values ranging from 0.0% in Rincon de Osa to 68.6% in Punta Banco (Table 3). This variation in Bd prevalence was best explained by the interaction effects model (Table 4), which showed significant effects of locality (*p* < 0.01), and that the variation of Bd prevalence by site depends on the habitat (*p* < 0.001; Figure 3a). Despite being close in proximity, amphibian assemblages from Sarapiqui showed significant higher prevalence of Bd than assemblages from Siquirres (*p* < 0.01, Figure 3a, Tables 3 and 5). There was a nonsignificant trend for Bd prevalence to be lower in Siquirres than Punta Banco (*p* = 0.06). We also found high prevalence of Bd across habitats (Table 3), but no significant differences between habitats in our model (*p* = 0.20).

**Table 3:** Prevalence (95% CI) and infection intensity (*SE*) of *Batrachochytrium dendrobatidis* in amphibian assemblages from four lowland sites and three lowland habitats of Costa Rica

Predictors		*n*	Prevalence (95% CI)	Infection intensity (*SE*)
Site	Rincon de Osa	25	0.0 (0.0–13.7)	0 (0.0)
	Punta Banco	35	68.6 (50.7–83.2)	2.0 (0.2)
	Sarapiqui	144	67.4 (51.1–75.5)	0.9 (0.1)
	Siquirres	144	47.9 (39.5–56.4)	1.5 (0.1)
Habitat	Forest	150	62.7 (54.4–70.4)	1.2 (0.1)
	Pond	116	39.7 (30.7–49.2)	1.3 (0.1)
	Stream	82	61.0 (49.6–71.6)	1.2 (0.2)

**Table 4:** Candidacy generalized linear models (GLMs) and linear models (LMs) used to determine the best predictors of prevalence of *Batrachochytrium dendrobatidis* and infection intensity in amphibian assemblages from four lowland sites and three lowland reproductive habitats in four lowland sites of Costa Rica

Model	AIC (GLMs)	*R* ^2^ (LMs)
Site*habitat (interaction model)	422.03	0.19
Site + habitat (additive model)	431.40	0.14
Site	432.90	0.13
Habitat	469.70	0.00

Note

The most robust models were selected according the highest values for the Akaike information criteria (AIC) for the generalized linear models (GLMs) and the coefficient of regression (*R* ^2^) for the linear models (LMs).

**Table 5:** Matrix of pairwise comparisons showing *p* values obtained from a post hoc analysis (Tukey test) to explain prevalence and infection intensity of *Batrachochytrium dendrobatidis* in amphibian assemblages from four lowland sites of Costa Rica

	Punta Banco	Sarapiqui	Siquirres
Bd prevalence			
Punta Banco			
Sarapiqui	0.98		
Siquirres	0.06	*p* < 0.01	
Bd Infection intensity			
Punta Banco			
Sarapiqui	*p* < 0.001		
Siquirres	0.12	*p* < 0.001	

Note

The table does not show results for Rincon de Osa because Bd prevalence at that site was 0%.

Similarly, the differences in the infection intensity across study sites (Figure 3b, Table 3) were best explained by the interaction model (*R* ^2 ^= 0.19, Table 4), which also showed significant effects of location (*F*_2,166_ = 15.5, *p* < 0.001) and the interaction between habitat and location (*F*_3,166_ = 3.6, *p* < 0.01). Levels of infection intensity were significantly lower in Sarapiqui (Figure 3b, Tables 3 and 5) when compared to Punta Banco (*p* < 0.001) and Siquirres (*p* < 0.01). Overall, the infection intensity ranged from 0.1 to 63,861 genome equivalents and four individuals had more than 10,000 zoospore genomic equivalents, a theoretical number that is considered a threshold that results in mass mortality and rapid population decline (Vredenburg et al., 2010). However, none of the sampled individuals including the four that were heavily infected showed any evident signs of disease. Remarkably, three of these heavily infected individuals belong to the Critically Endangered species *Craugastor taurus*.

**Figure 4:**
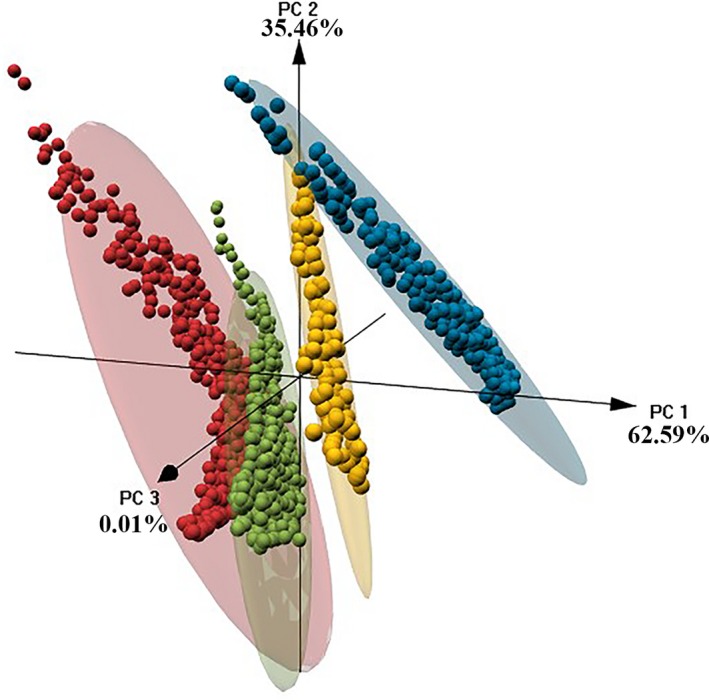
Abiotic environment of four surveyed lowland sites in Costa Rica. Tridimensional PCA biplot displays the extracted values within buffers (radius = 10 km) representing the four lowland sampling sites for the 19 bioclimatic variables from the WorldClim dataset

In our PCA analysis of the 19 bioclimatic variables, we retained the first two axes (Table 1) because they accounted for 98% of the total variance of our data. A tridimensional representation of PCA axes 1 and 2 (PCA 3 included as reference) shows four separated clusters of points, each one representing a study site (Figure 4). As expected, we found the highest similarity in climatic conditions occurred among sites in each zone (Appendix A). We found that bioclimatic variables associated with precipitation (Annual Precipitation, Precipitation of Wettest Quarter, Precipitation of Driest Quarter) make a higher contribution in the variance of our climatic data than other variables (Table 1).

## 4 DISCUSSION

We found Bd infections at three of the four lowland sites sampled and in 70.2% of the 47 sampled species for an overall Bd prevalence of 54.6% (Tables 2 and 3). Furthermore, we did not detect signs of disease in heavily infected individuals during the study and found low levels of infection in most of our samples. Similar community composition and population dynamics observed during our study and later visits (unpublished data) suggest that host–pathogen dynamics in surveyed lowlands are exhibiting enzootic dynamics, rather than epizootic dynamics (Brem & Lips, 2008; Briggs, Knapp, & Vredenburg, 2010; Perez et al., 2014; Woodhams et al., 2008). Our findings also suggest that the distribution of Bd in Costa Rica is wider than historically considered (Puschendorf et al., 2009) and that the population declines during the 1980s and 1990s may not have been restricted to highlands. Comparable results were found in lowlands of Panama where Bd has been detected in multiple lowland sites (Kilburn et al., 2010; Perez et al., 2014; Woodhams et al., 2008). We suggest that future studies should include replicated sampling across seasons and sites that are outside the optimal environmental conditions for Bd growth, especially since most of these optimal conditions been estimated from lab studies. Additionally, under potential scenarios of climate change, sites that are currently considered unsuitable for Bd may experience future outbreaks of chytridiomycosis if environmental conditions become closer to ideal ranges for Bd growth (AlMutairi, Grossmann, & Small, 2019; Enquist, 2002). Furthermore, conducting more studies and replicated samplings in neglected sites or locations that are assumed to be pathogen‐free may help to better describe spatial dynamics of both the host and pathogen. These proposed studies could reduce the effect of opportunistically collected data from montane ecosystems and help develop more effective conservation tools and actions for amphibians in a broader range of habitats (Garner et al., 2016; Grenyer et al., 2006; Scheele et al., 2014; Woodhams et al., 2011).

The three endangered species sampled (*Craugastor taurus, Agalychnis lemur,* and *Oophaga granulifera*) tested positive for Bd (Table 2). The populations of *C. taurus* and *A. lemur* that we surveyed also tested positive in past surveys (Briggs et al., 2010; Whitfield et al., 2017). The continuous occurrence of these endangered species and the lack of clinical signs of chytridiomycosis in Bd‐infected individuals (Berger et al., 1998; Voyles et al., 2009) suggest these populations are capable of surviving with enzootic Bd dynamics (Whitfield et al., 2017). Remarkably, infection levels in several individuals of the robber frog (*C. taurus*) were above 10,000 Bd genomic equivalents, a theoretical threshold that has been linked to epizootic outbreaks, population die‐offs, and local extinctions (Vredenburg et al., 2010). There are several explanations for these high infection loads without signs of population decline or disease. For example, it is possible that these populations can coexist with Bd because they carry cutaneous bacteria that release anti‐Bd compounds, although none have been detected in individuals of the relict populations of the Golfito robber frog (Madison et al., 2017) or in a similar critically endangered species (*C. ranoides*) which also catastrophically declined in the 1980s (Puschendorf et al., 2009; Zumbado‐Ulate, Bolaños, Willink, & Soley‐Guardia, 2011). Additionally, antimicrobial peptides and immune defenses (innate and adaptive) may play a role in this host–pathogen coexistence (Rollins‐Smith, 2017; Woodhams et al., 2016). Alternatively, persistence of these populations could be associated with behavioral adaptations that rapidly clear infection or to local dry conditions that constrain Bd growth allowing susceptible frogs to coexist with low levels of Bd infection (Chaves et al., 2014; Puschendorf et al., 2011). Further studies on these endangered lowland populations can lead to management plans that protect and stabilize these relict populations.

The absence of Bd in fourteen surveyed species could be an artifact of the small sample sizes (1–10 individuals, Table 2) because some of these species have tested positive in other studies in Costa Rica and nearby Panama (e.g., *Engystomops pustulosus*, *Duellmanohyla rufioculis*, *Anotheca spinosa*, *Leptodactylus poecilochilus*) (Picco & Collins, 2007; Rodríguez‐Brenes, Rodriguez, Ibáñez, & Ryan, 2016; Zumbado‐Ulate et al., 2014). Low sample sizes were caused by low detectability during the survey period for some of the common species (e.g., *Rhinella horribilis,* *Smilisca sordida, Lithobates vaillanti, Leptodactylus savagei*) or due to the low year‐round detectability for the more cryptic and rare species (e.g., fossorial and canopy dwellers like *Oedipina gracilis, Bolitoglossa colonnea, Cruziohyla calcarifer*). To increase species detectability and/or sample size, future studies in lowlands and neglected sites should conduct surveys restricting or focusing the sampling on threatened species (Thorpe et al., 2018; Whitfield et al., 2017), to describe host–pathogen population dynamics, or preferably survey multiple species across seasons to obtain more accurate estimates of prevalence and infection intensity for all species within the amphibian community (Brannelly et al., 2015; Kinney et al., 2011; Vredenburg et al., 2010).

We found common lowland species with high prevalence of Bd (e.g., *Lithobates warszewithschii, Craugastor fitzingeri, Rhaebo haematiticus, Oophaga pumilio, Dendropsophus ebraccatus*). The species *L. warszewithschii, C. fitzingeri, *and *D. ebraccatus* also inhabit the montane ecosystems where historical enigmatic declines occurred. These species and others not sampled here (e.g., *Isthmohyla pseudopuma*) or with a small sample size (e.g., *Smilisca sordida*) seem to be highly tolerant to Bd and may function as competent reservoirs (Ostfeld & Keesing, 2000; Reeder, Pessier, & Vredenburg, 2012; Scheele et al., 2017), amplifying Bd infection in the community (Searle, Biga, Spatafora, & Blaustein, 2011). Therefore, the high infection prevalence in these species that we found at lowland sites suggests that Bd is common and persists in these locations.

Our results showed that Bd was widespread across lowlands during the time of study, but Bd prevalence and intensity might exhibit seasonal dynamics. However, to detect a seasonality effect, multi‐season studies collecting samples from a variety of amphibian assemblages must be conducted (Kinney et al., 2011; Phillott et al., 2013; Savage et al., 2011). Similar studies conducted in lowlands of Costa Rica also suggest seasonal dynamics. For example, remnant populations of the lowland robber frog *C. ranoides* in the tropical dry forest of Costa Rica exhibited infection prevalence values that varied from <1 to 60% across a dry season (December to May) (Whitfield et al., 2017; Zumbado‐Ulate et al., 2014). Similarly, prevalence of Bd varied from <5% to around 35% in an amphibian assemblage in tropical lowland forest across 1‐year period (Whitfield et al., 2012). Therefore, follow‐up studies across lowlands in Costa Rica are needed to identify seasonal dynamics of Bd in Costa Rica, which may help design more suitable conservation strategies for lowlands endangered populations.

We did not find Bd in our samples from Rincon de Osa, and a similar study also reported a very low prevalence of Bd in the same study sites and nearby zones across the Osa Peninsula (Goldberg, Hawley, & Waits, 2009). Although our detected prevalence in Rincon de Osa was 0%, our binomial confidence interval (0%–95%) overlaps with the prevalence value presented in this study. Therefore, our result for Rincon de Osa might be an artifact of our low sample size (*n* = 24) which is not large enough to achieve 95% certainty of detecting 1 positive individual, based on the minimum disease prevalence of ≥5% in infected amphibian assemblages (Skerratt et al., 2008). Climatic conditions at Rincon de Osa might constrain the dispersal and growth of Bd allowing coexistence between susceptible frogs and Bd (i.e., environmental refuge from chytridiomycosis, Puschendorf et al., 2011). However, the extirpation of the Golfito robber frog in this area, where it was abundant before the 1980s and 1990s (Chaves et al., 2014), suggests this may not be the case. We also found the highest levels of Bd prevalence in the Caribbean sites which coincide with studies conducted in the nearby locations within the same geographic zone (Whitfield et al., 2017, 2013, 2012). Thus, even within lowland zones, there is large variation in Bd prevalence across zones and sites.

Our statistical models showed no differences among habitats in relation to prevalence and infection intensity. However, there was a trend for higher prevalence of Bd in forest assemblages than in aquatic assemblages (lotic and lentic, Table 3), which differs from other similar studies (Brem & Lips, 2008; Kriger & Hero, 2007a; Lips et al., 2003). Some of the sampled species (e.g., *Craugastor fitzingeri, Oophaga pumilio, Rhaebo haematiticus, Rhinella horribilis*) may forage or move through different habitats that do not match their dwelling habitat, which may have affected our results. Previous studies have shown the highest infection prevalence and intensity in permanent streams, suggesting that continuous streamflow provides more suitable conditions for the spread of Bd than other habitats (Kriger & Hero, 2007a; Lips et al., 2003). Lentic environments are more exposed to sunlight, resulting in temperatures >30°C (Adams et al., 2017), which in laboratory conditions is unsuitable for Bd (Piotrowski et al., 2004). Lentic environments also sustain invertebrates that feed on zoospores reducing the proportion of infected individuals (e.g., *Daphnia* spp.*,* Searle, Mendelson, Green, & Duffy, 2013). However, our findings suggest that the role of terrestrial lowland ecosystems in the dispersal of Bd might have been underestimated. (but see Whitfield et al., 2012; Whitfield et al., 2013). Therefore, multiseason studies contrasting Bd dynamics across habitats are needed to elucidate the role of microhabitats in sustaining Bd.

We found significant evidence that every site of study represents an independent local abiotic environment according to the 19 environmental predictors that we used in our analysis (Figure 4). This climatic independence was consistent with the heterogeneous prevalence of Bd, which suggests that every site exhibits a different host–pathogen dynamic in response to local environmental conditions. However, irregularity in elevation gradient across our study sites (Kriger & Hero, 2008), especially in the study site of Siquirres, where elevations varied from 400 to 600 m, could have influenced the differential prevalence we found across lowlands. We recommend controlling for elevational gradients (Kilburn et al., 2010) in follow‐up studies. Seasonality and particularly differences in precipitation (Table 1) may also play an important role in differential Bd prevalence between the Caribbean and South Pacific zones. The south Pacific zone, where Punta Banco and Rincon de Osa occur, presents a dry season extending from December to April, which coincided with our sampling. Conversely, the Caribbean zone does not have a well‐established dry season, and the rainy season starts in December, when we conducted our surveys (Herrera, 1985). Other studies conducted at larger scale have also shown seasonal and latitudinal variation of Bd prevalence and infection (Brannelly et al., 2015; Kinney et al., 2011; Kriger et al., 2007; Phillott et al., 2013). Future studies should evaluate the effect of elevational gradients on the amphibian host‐Bd dynamics.

Our results suggest that researchers should expand their sampling across the entire distribution of focal species and communities instead of only focusing on sites of historical declines. An adequate seasonal description of the suitable abiotic environment of pathogens across the host amphibian home range may help identify disease‐free sites for effective repatriation or to determine instances were more technical strategies are needed to secure maintenance of declined populations (e.g., antifungal treatments to clear infection, bioaugmentation with commensal bacteria, habitat manipulation, ex‐situ conservation) (Garner et al., 2016; Scheele et al., 2014). Furthermore, conducting more seasonal sampling in lowlands will increase the record of presence–absence datasets on Bd and can be used to generate more robust species distribution models (SDMs) from nonopportunistically collected data (Puschendorf et al., 2013). SDMs can help identify hotspots for future outbreaks of Bd and can be used to predict potential locations for amphibian rediscoveries (García‐Rodríguez et al., 2012; Puschendorf et al., 2009). Recent validation surveys have led to the discovery of relict peripheral populations that occur in potential environmental refuges from disease (Puschendorf et al., 2011; Raffel & Fox, 2018; Scheele, Hunter, Skerratt, Brannelly, & Driscoll, 2015), validating increased surveys outside the boundaries of core geographic distributions (Abarca, Chaves, García‐Rodríguez, & Vargas, 2010; Chaves et al., 2014; González‐Maya et al., 2013; Jiménez & Alvarado, 2017; Nishida, 2006). A comprehensive assessment of a pathogen's distribution, prevalence, and infection intensity can lead to more effective disease‐management strategies based on specific locations, habitats, and species.

## CONFLICT OF INTEREST

The authors declare no conflict of interest exists.

## AUTHOR CONTRIBUTIONS

HZ‐U, AG‐R, CS, and VV developed the ideas and designed methodology; HZ‐U and AG‐R conducted data collection and data analysis; HZ‐U, AG‐R, CS, and VV led the writing of the manuscript. All authors contributed critically to the drafts and gave final approval for publication.

## Data Availability

The data associated with this publication (Data files title: Bd_lowlands_Costa_Rica) are deposited at Dryad data repository. Provisional https://doi.org/10.5061/dryad.8t267j0.

## APPENDIX A

Cluster analysis of four lowland sites in Costa Rica generated from a matrix of Euclidean distances between the centroids of climatic envelopes. The cluster shows higher similarities between Sarapiqui and Siquirres (Caribbean side) and Rincon de Osa‐Punta Banco (Pacific side)

## References

[ece35098-bib-0001] Abarca, J. , Chaves, G. , García‐Rodríguez, A. , & Vargas, R. (2010). Reconsidering extinction: Rediscovery of *Incilius holdridgei* (Anura: Bufonidae) in Costa Rica after 25 years. Herpetological Review, 41, 150.

[ece35098-bib-0002] Adams, A. J. , Kupferberg, S. J. , Wilber, M. Q. , Pessier, A. P. , Grefsrud, M. , Bobzien, S. , … Briggs, C. J. (2017). Extreme drought, host density, sex, and bullfrogs influence fungal pathogen infection in a declining lotic amphibian. Ecosphere, 8, e01740 10.1002/ecs2.1740

[ece35098-bib-0003] AlMutairi, B. S. , Grossmann, I. , & Small, M. J. (2019). Climate model projections for future seasonal rainfall cycle statistics in Northwest Costa Rica. International Journal of Climatology, 2019, 4917–114. 10.1002/joc.5993

[ece35098-bib-0004] Barnosky, A. D. , Matzke, N. , Tomiya, S. , Wogan, G. O. U. , Swartz, B. , Quental, T. B. , … Ferrer, E. A. (2011). Has the Earth's sixth mass extinction already arrived? Nature, 471, 51–57. 10.1038/nature09678 21368823

[ece35098-bib-0005] Berger, L. , Speare, R. , Daszak, P. , Green, D. E. , Cunningham, A. A. , Goggin, C. l. , … Parkes, H. (1998). Chytridiomycosis causes Amphibian mortality associated with population declines in the rain forests of Australia and Central America. Proceedings of the National Academy of Sciences of the United States of America, 95, 9031–9036. 10.1073/pnas.95.15.9031 9671799 PMC21197

[ece35098-bib-0006] Berger, L. , Speare, R. , Hines, H. B. , Marantelli, G. , Hyatt, A. D. , McDonald, K. R. , … Tyler, M. J. (2004). Effect of season and temperature on mortality in amphibians due to chytridiomycosis. Australian Veterinary Journal, 82, 434–439. 10.1111/j.1751-0813.2004.tb11137.x 15354853

[ece35098-bib-0007] Boyle, D. G. , Boyle, D. B. , Olsen, V. , Morgan, J. A. T. , & Hyatt, A. D. (2004). Rapid quantitative detection of chytridiomycosis (*Batrachochytrium dendrobatidis*) in amphibian samples using real‐time Taqman PCR assay. Diseases of Aquatic Organisms, 60, 141–148. 10.3354/dao060141 15460858

[ece35098-bib-0008] Brannelly, L. A. , Hunter, D. A. , Lenger, D. , Scheele, B. C. , Skerratt, L. F. , & Berger, L. (2015). Dynamics of chytridiomycosis during the breeding season in an Australian Alpine amphibian. PLoS One, 10, e0143629 10.1371/journal.pone.0143629 26629993 PMC4668081

[ece35098-bib-0009] Brannelly, L. A. , Martin, G. , Llewelyn, J. , Skerratt, L. F. , & Berger, L. (2018). Age‐and size‐dependent resistance to chytridiomycosis in the invasive cane toad *Rhinella marina* . Diseases of Aquatic Organisms, 131, 107–120. 10.3354/dao03278 30460917

[ece35098-bib-0010] Brem, F. , & Lips, K. (2008). *Batrachochytrium* *dendrobatidis* infection patterns among Panamanian amphibian species, habitats and elevations during epizootic and enzootic stages. Diseases of Aquatic Organisms, 81, 189–202. 10.3354/dao01960 18998584

[ece35098-bib-0011] Briggs, C. J. , Knapp, R. A. , & Vredenburg, V. T. (2010). Enzootic and epizootic dynamics of the chytrid fungal pathogen of amphibians. Proceedings of the National Academy of Sciences of the United States of America, 107, 9695–9700. 10.1073/pnas.0912886107 20457916 PMC2906864

[ece35098-bib-0012] Brooks, T. M. , Mittermeier, R. A. , da Fonseca, G. A. B. , Gerlach, J. , Hoffmann, M. , Lamoreux, J. F. , … Rodrigues, A. S. L. (2006). Global biodiversity conservation priorities. Science, 313, 58–61. 10.1126/science.1127609 16825561

[ece35098-bib-0013] Burnham, K. P. , & Anderson, D. R. (2004). Multimodel inference: Understanding AIC and BIC in model selection. Sociological Methods & Research, 33, 61–304. 10.1177/0049124104268644

[ece35098-bib-0014] Catenazzi, A. (2015). State of the world's amphibians. Annual Review of Environment and Resources, 40, 91–119. 10.1146/annurev-environ-102014-021358

[ece35098-bib-0015] Chaves, G. , Zumbado‐Ulate, H. , García‐Rodríguez, A. , Gómez, E. , Vredenburg, V. T. , & Ryan, M. J. (2014). Rediscovery of the critically endangered streamside frog, *Craugastor taurus *(Craugastoridae), in Costa Rica. Tropical Conservation Science, 7, 628–638. 10.1177/194008291400700404

[ece35098-bib-0016] Cheng, T. L. , Rovito, S. M. , Wake, D. B. , & Vredenburg, V. T. (2011). Coincident mass extirpation of neotropical amphibians with the emergence of the infectious fungal pathogen *Batrachochytrium* *dendrobatidis* . Proceedings of the National Academy of Sciences of the United States of America, 108, 9502–9507. 10.1073/pnas.1105538108 21543713 PMC3111304

[ece35098-bib-0017] Collins, J. P. (2010). Amphibian decline and extinction: What we know and what we need to learn. Diseases of Aquatic Organisms, 92, 93–99. 10.3354/dao02307 21268970

[ece35098-bib-0018] Enquist, C. A. F. (2002). Predicted regional impacts of climate change on the geographical distribution and diversity of tropical forests in Costa Rica. Journal of Biogeography, 29, 519–534. 10.1046/j.1365-2699.2002.00695.x

[ece35098-bib-0019] Fisher, M. C. , Garner, T. W. J. , & Walker, S. F. (2009). Global emergence of *Batrachochytrium dendrobatidis* and amphibian chytridiomycosis in space, time, and host. Annual Review of Microbiology, 63, 291–310. 10.1146/annurev.micro.091208.073435 19575560

[ece35098-bib-0020] Flechas, S. , Vredenburg, V. T. , & Amézquita, A. (2015). Infection prevalence in three lowland species of harlequin toads from the threatened genus *Atelopus* . Herpetological Review, 46, 528–532.

[ece35098-bib-0021] Foden, W. B. , Butchart, S. H. M. , Stuart, S. N. , Vié, J.‐C. , Akçakaya, H. R. , Angulo, A. , … Mace, G. M. (2013). Identifying the world's most climate change vulnerable species: A systematic trait‐based assessment of all birds, amphibians and corals. PLoS One, 8, e65427 10.1371/journal.pone.0065427 23950785 PMC3680427

[ece35098-bib-0022] García‐Rodríguez, A. , Chaves, G. , Benavides‐Varela, C. , & Puschendorf, R. (2012). Where are the survivors? Tracking relictual populations of endangered frogs in Costa Rica. Diversity and Distributions, 18, 204–212. 10.1111/j.1472-4642.2011.00862.x

[ece35098-bib-0023] Garner, T. W. J. , Schmidt, B. R. , Martel, A. , Pasmans, F. , Muths, E. , Cunningham, A. A. , … Bosch, J. (2016). Mitigating amphibian chytridiomycosis in nature. Philosophical Transactions of the Royal Society B: Biological Sciences, 371, 20160207 10.1098/rstb.2016.0207 PMC5095549 28080996

[ece35098-bib-0024] Giraudo, A. R. , & Arzamendia, V. (2018). Descriptive bioregionalisation and conservation biogeography: What is the true bioregional representativeness of protected areas? Australian Systematic Botany, 30, 403–413. 10.1071/SB16056

[ece35098-bib-0025] Goldberg, C. S. , Hawley, T. J. , & Waits, L. P. (2009). Local and regional patterns of amphibian chytrid prevalence on the Osa Peninsula, Costa Rica. Herpetological Review, 40, 309–311.

[ece35098-bib-0026] González‐Maya, J. F. , Belant, J. L. , Wyatt, S. A. , Schipper, J. , Cardenal, J. , Corrales, D. , … Fischer, A. (2013). Renewing hope: The rediscovery of *Atelopus varius* in Costa Rica. Amphibia‐Reptilia, 34, 573–578. 10.1163/15685381-00002910

[ece35098-bib-0027] Green, D. M. (2017). Amphibian breeding phenology trends under climate change: Predicting the past to forecast the future. Global Change Biology, 23, 646–656. 10.1111/gcb.13390 27273300

[ece35098-bib-0028] Grenyer, R. , Orme, C. D. L. , Jackson, S. F. , Thomas, G. H. , Davies, R. G. , Davies, T. J. , … Owens, I. P. F. (2006). Global distribution and conservation of rare and threatened vertebrates. Nature, 444, 93–96. 10.1038/nature05237 17080090

[ece35098-bib-0029] Hero, J.‐M. , Williams, S. E. , & Magnusson, W. E. (2005). Ecological traits of declining amphibians in upland areas of eastern Australia. Journal of Zoology, 267, 221–232. 10.1017/S0952836905007296

[ece35098-bib-0030] Herrera, W. (1985). Clima de Costa Rica (Vegetación y clima de Costa Rica, Vol. 2). Universidad Estatal a Distancia.

[ece35098-bib-0031] Heyer, W. R. , Donnelly, M. A. , McDiarmid, R. A. , Hayek, L. C. , & Foster, M. S. (Eds.) (1994). Measuring and monitoring biological diversity: Standard methods for amphibians. Washington, DC: Smithsonian Institution Press.

[ece35098-bib-0032] Hijmans, R. J. , Cameron, S. E. , Parra, J. L. , Jones, P. G. , & Jarvis, A. (2005). Very high resolution interpolated climate surfaces for global land areas. International Journal of Climatology, 25, 1965–1978. 10.1002/joc.1276

[ece35098-bib-0033] Hirschfeld, M. , Blackburn, D. C. , Doherty‐Bone, T. M. , Gonwouo, L. N. , Ghose, S. , & Rödel, M.‐O. (2016). Dramatic declines of montane frogs in a Central African biodiversity hotspot. PLoS One, 11, e0155129 10.1371/journal.pone.0155129 27149624 PMC4858272

[ece35098-bib-0034] Hitchman, S. M. , Mather, M. E. , Smith, J. M. , & Fencl, J. S. (2018). Identifying keystone habitats with a mosaic approach can improve biodiversity conservation in disturbed ecosystems. Global Change Biology, 24, 308–321. 10.1111/gcb.13846 28755429

[ece35098-bib-0035] Holdridge, L. R. (1967). Life zone ecology. Tropical Science Center.

[ece35098-bib-0036] Hooper, D. U. , Adair, E. C. , Cardinale, B. J. , Byrnes, J. E. K. , Hungate, B. A. , Matulich, K. L. , … O'Connor, M. I. (2012). A global synthesis reveals biodiversity loss as a major driver of ecosystem change. Nature, 486, 105–108. 10.1038/nature11118 22678289

[ece35098-bib-0037] James, T. Y. , Toledo, L. F. , Rödder, D. , da Silva Leite, D. , Belasen, A. M. , Betancourt‐Román, C. M. , … Longcore, J. E. (2015). Disentangling host, pathogen, and environmental determinants of a recently emerged wildlife disease: Lessons from the first 15 years of amphibian chytridiomycosis research. Ecology and Evolution, 5, 4079–4097. 10.1002/ece3.1672 26445660 PMC4588650

[ece35098-bib-0038] Jiménez, R. , & Alvarado, G. (2017). *Craugastor escoces* (Anura: Craugastoridae) reappears after 30 years: Rediscovery of an “extinct” Neotropical frog. Amphibia‐Reptilia, 38, 257–259. 10.1163/15685381-00003102

[ece35098-bib-0039] Kilburn, V. L. , Ibáñez, R. , Sanjur, O. , Bermingham, E. , Suraci, J. P. , & Green, D. M. (2010). Ubiquity of the pathogenic chytrid fungus, Batrachochytrium dendrobatidis, in anuran communities in Panamá. EcoHealth, 7, 537–548. 10.1007/s10393-010-0634-1 21225313

[ece35098-bib-0040] Kinney, V. C. , Heemeyer, J. L. , Pessier, A. P. , & Lannoo, M. J. (2011). Seasonal pattern of *Batrachochytrium dendrobatidis* infection and mortality in *Lithobates areolatus*: Affirmation of Vredenburg's “10,000 zoospore rule”. PLoS One, 6, e16708 10.1371/journal.pone.0016708 21423745 PMC3053364

[ece35098-bib-0041] Kriger, K. M. , & Hero, J.‐M. (2007a). The chytrid fungus *Batrachochytrium dendrobatidis* is non‐randomly distributed across amphibian breeding habitats. Diversity and Distributions, 13, 781–788. 10.1111/j.1472-4642.2007.00394.x

[ece35098-bib-0042] Kriger, K. M. , & Hero, J.‐M. (2007b). Large‐scale seasonal variation in the prevalence and severity of chytridiomycosis. Journal of Zoology, 271, 352–359. 10.1111/j.1469-7998.2006.00220.x

[ece35098-bib-0043] Kriger, K. M. , & Hero, J.‐M. (2008). Altitudinal distribution of chytrid (*Batrachochytrium* *dendrobatidis*) infection in subtropical Australian frogs. Austral Ecology, 33, 1022–1032. 10.1111/j.1442-9993.2008.01872.x

[ece35098-bib-0044] Kriger, K. M. , Hero, J.‐M. , & Ashton, K. J. (2006). Cost efficiency in the detection of chytridiomycosis using PCR assay. Diseases of Aquatic Organisms, 71, 149–154. 10.3354/dao071149 16956062

[ece35098-bib-0045] Kriger, K. M. , Hines, H. B. , Hyatt, A. D. , Boyle, D. G. , & Hero, J.‐M. (2006). Techniques for detecting chytridiomycosis in wild frogs: Comparing histology with real‐time TaqMan PCR. Diseases of Aquatic Organisms, 71, 141–148. 10.3354/dao071141 16956061

[ece35098-bib-0046] Kriger, K. M. , Pereoglou, F. , & Hero, J.‐M. (2007). Latitudinal variation in the prevalence and intensity of chytrid (*Batrachochytrium dendrobatidis*) infection in Eastern Australia. Conservation Biology, 21, 1280–1290. 10.1111/j.1523-1739.2007.00777.x 17883493

[ece35098-bib-0047] La Marca, E. , Lips, K. R. , Lotters, S. , Puschendorf, R. , Ibanez, R. , Rueda‐Almonacid, J. V. , … Young, B. E. (2005). Catastrophic population declines and extinctions in Neotropical harlequin frogs (Bufonidae: *Atelopus*). Biotropica, 37, 190–201. 10.1111/j.1744-7429.2005.00026.x

[ece35098-bib-0048] Lips, K. R. (1998). Decline of a tropical montane amphibian fauna. Conservation Biology, 12, 106–117. 10.1111/j.1523-1739.1998.96359.x

[ece35098-bib-0049] Lips, K. R. (1999). Mass mortality and population declines of anurans at an upland site in western Panama. Conservation Biology, 13, 117–125. 10.1046/j.1523-1739.1999.97185.x

[ece35098-bib-0050] Lips, K. R. , Diffendorfer, J. , Mendelson, J. R. III , & Sears, M. W. (2008). Riding the wave: Reconciling the roles of disease and climate change in amphibian declines. PLOS Biology, 6, e72 10.1371/journal.pbio.0060072 18366257 PMC2270328

[ece35098-bib-0051] Lips, K. R. , Reeve, J. D. , & Witters, L. R. (2003). Ecological traits predicting amphibian population declines in Central America. Conservation Biology, 17, 1078–1088. 10.1046/j.1523-1739.2003.01623.x

[ece35098-bib-0052] Longcore, J. E. , Pessier, A. P. , & Nichols, D. K. (1999). *Batrachochytrium dendrobatidis* gen. et sp. nov. a chytrid pathogenic to amphibians. Mycologia, 91, 219–227. 10.2307/3761366

[ece35098-bib-0053] Madison, J. D. , Berg, E. A. , Abarca, J. G. , Whitfield, S. M. , Gorbatenko, O. , Pinto, A. , & Kerby, J. L. (2017). Characterization of *Batrachochytrium dendrobatidis* inhibiting bacteria from amphibian populations in Costa Rica. Frontiers in Microbiology, 8, 290 10.3389/fmicb.2017.00290 28293222 PMC5329008

[ece35098-bib-0054] Miller, C. A. , Tasse Taboue, G. C. , Ekane, M. M. P. , Robak, M. , Sesink Clee, P. R. , Richards‐Zawacki, C. , … Anthony, N. M. (2018). Distribution modeling and lineage diversity of the chytrid fungus *Batrachochytrium dendrobatidis* (Bd) in a central African amphibian hotspot. PLoS One, 13, e0199288 10.1371/journal.pone.0199288 29924870 PMC6010240

[ece35098-bib-0055] Monastersky, R. (2014). Life‐a status report. Nature, 516, 159–161. 10.1038/516158a 25503217

[ece35098-bib-0056] Murray, K. A. , Retallick, R. W. , Puschendorf, R. , Skerratt, L. F. , Rosauer, D. , McCallum, H. I. , … VanDerWal, J. (2011). Assessing spatial patterns of disease risk to biodiversity: Implications for the management of the amphibian pathogen, *Batrachochytrium dendrobatidis* . Journal of Applied Ecology, 48, 163–173. 10.1111/j.1365-2664.2010.01890.x

[ece35098-bib-0057] Nishida, K. (2006). Encounter with *Hyla angustilineata* Taylor, 1952 (Anura: Hylidae) in a cloud forest of Costa Rica. Brenesia, 66, 79–81.

[ece35098-bib-0058] Novacek, M. J. , & Cleland, E. E. (2001). The current biodiversity extinction event: Scenarios for mitigation and recovery. Proceedings of the National Academy of Sciences of the United States of America, 98, 5466–5470. 10.1073/pnas.091093698 11344295 PMC33235

[ece35098-bib-0059] Nowakowski, A. J. , Whitfield, S. M. , Eskew, E. A. , Thompson, M. E. , Rose, J. P. , Caraballo, B. L. , … Todd, B. D. (2016). Infection risk decreases with increasing mismatch in host and pathogen environmental tolerances. Ecology Letters, 19, 1051–1061. 10.1111/ele.12641 27339786

[ece35098-bib-0060] Olson, D. H. , Aanensen, D. M. , Ronnenberg, K. L. , Powell, C. I. , Walker, S. F. , Bielby, J. , … Fisher, M. C. (2013). Mapping the global emergence of *Batrachochytrium dendrobatidis*, the amphibian chytrid fungus. PLoS One, 8, e56802 10.1371/journal.pone.0056802 23463502 PMC3584086

[ece35098-bib-0061] Ostfeld, R. S. , & Keesing, F. (2000). Biodiversity and disease risk: The case of Lyme disease. Conservation Biology, 14, 722–728. 10.1046/j.1523-1739.2000.99014.x

[ece35098-bib-0062] Perez, R. , Richards‐Zawacki, C. L. , Krohn, A. R. , Robak, M. , Griffith, E. J. , Ross, H. , … Voyles, J. (2014). Field surveys in Western Panama indicate populations of *Atelopus varius* frogs are persisting in regions where *Batrachochytrium dendrobatidis* is now enzootic. Amphibian & Reptile Conservation, 8, 30–35.

[ece35098-bib-0063] Phillott, A. D. , Grogan, L. F. , Cashins, S. D. , Mcdonald, K. R. , Berger, L. , & Skerratt, L. F. (2013). Chytridiomycosis and seasonal mortality of tropical stream‐associated frogs 15 years after introduction of *Batrachochytrium dendrobatidis* . Conservation Biology, 27, 1058–1068. 10.1111/cobi.12073 23678872

[ece35098-bib-0064] Picco, A. M. , & Collins, J. P. (2007). Fungal and viral pathogen occurrence in Costa Rican amphibians. Journal of Herpetology, 41, 746–749. 10.1670/07-033.1

[ece35098-bib-0065] Piotrowski, J. S. , Annis, S. L. , & Longcore, J. E. (2004). Physiology of *Batrachochytrium* *dendrobatidis*, a chytrid pathogen of amphibians. Mycologia, 96, 9–15. 10.1080/15572536.2005.11832990 21148822

[ece35098-bib-0066] Pounds, J. A. , Bustamante, M. R. , Coloma, L. A. , Consuegra, J. A. , Fogden, M. P. L. , Foster, P. N. , … Young, B. E. (2006). Widespread amphibian extinctions from epidemic disease driven by global warming. Nature, 439, 161–167. 10.1038/nature04246 16407945

[ece35098-bib-0067] Pounds, J. A. , & Crump, M. L. (1994). Amphibian declines and climate disturbance: The case of the golden toad and the harlequin frog. Conservation Biology, 8, 72–85. 10.1046/j.1523-1739.1994.08010072.x

[ece35098-bib-0068] Puschendorf, R. , Bolaños, F. , & Chaves, G. (2006). The amphibian chytrid fungus along an altitudinal transect before the first reported declines in Costa Rica. Biological Conservation, 132, 36–142. 10.1016/j.biocon.2006.03.010

[ece35098-bib-0069] Puschendorf, R. , Carnaval, A. C. , VanDerWal, J. , Zumbado‐Ulate, H. , Chaves, G. , Bolaños, F. , & Alford, R. A. (2009). Distribution models for the amphibian chytrid *Batrachochytrium dendrobatidis* in Costa Rica: Proposing climatic refuges as a conservation tool. Diversity and Distributions, 15, 401–408. 10.1111/j.1472-4642.2008.00548.x

[ece35098-bib-0070] Puschendorf, R. , Hodgson, L. , Alford, R. A. , Skerratt, L. F. , & VanDerWal, J. (2013). Underestimated ranges and overlooked refuges from amphibian chytridiomycosis. Diversity and Distributions, 19, 1313–1321. 10.1111/ddi.12091

[ece35098-bib-0071] Puschendorf, R. , Hoskin, C. J. , Cashins, S. D. , McDonald, K. , Skerratt, L. F. , Vanderwal, J. , & Alford, R. A. (2011). Environmental refuge from disease‐driven amphibian extinction. Conservation Biology, 25, 956–964. 10.1111/j.1523-1739.2011.01728.x 21902719

[ece35098-bib-0072] R Core Team (2014). R: The R project for statistical computing. Retrieved from http://www.R-project.org/

[ece35098-bib-0073] Raffel, T. R. , & Fox, C. (2018). How do newts fight disease? They change their habitat. Functional Ecology, 32, 1142–1144. 10.1111/1365-2435.13111

[ece35098-bib-0074] Reeder, N. M. , Pessier, A. P. , & Vredenburg, V. T. (2012). A reservoir species for the emerging amphibian pathogen *Batrachochytrium dendrobatidis* thrives in a landscape decimated by disease. PLoS One, 7, e33567 10.1371/journal.pone.0033567 22428071 PMC3299797

[ece35098-bib-0075] Retallick, R. W. , McCallum, H. , & Speare, R. (2004). Endemic infection of the amphibian chytrid fungus in a frog community post‐decline. PLOS Biology, 2, e351 10.1371/journal.pbio.0020351 15502873 PMC521176

[ece35098-bib-0076] Rödder, D. , Kielgast, J. , & Lötters, S. (2010). Future potential distribution of the emerging amphibian chytrid fungus under anthropogenic climate change. Diseases of Aquatic Organisms, 92, 201–207. 10.3354/dao02197 21268982

[ece35098-bib-0077] Rödder, D. , Veith, M. , & Lötters, S. (2008). Environmental gradients explaining the prevalence and intensity of infection with the amphibian chytrid fungus: The host's perspective. Animal Conservation, 11, 513–517. 10.1111/j.1469-1795.2008.00210.x

[ece35098-bib-0078] Rodríguez‐Brenes, S. , Rodriguez, D. , Ibáñez, R. , & Ryan, M. J. (2016). Spread of amphibian chytrid fungus across lowland populations of túngara frogs in Panamá. PLoS One, 11, e0155745 10.1371/journal.pone.0155745 27176629 PMC4866759

[ece35098-bib-0079] Rollins‐Smith, L. A. (2017). Amphibian immunity–stress, disease, and climate change. Developmental & Comparative Immunology, 66, 111–119. 10.1016/j.dci.2016.07.002 27387153

[ece35098-bib-0080] Rosenblum, E. B. , James, T. Y. , Zamudio, K. R. , Poorten, T. J. , Ilut, D. , Rodriguez, D. , … Stajich, J. E. (2013). Complex history of the amphibian‐killing chytrid fungus revealed with genome resequencing data. Proceedings of the National Academy of Sciences of the United States of America, 110, 9385–9390. 10.1073/pnas.1300130110 23650365 PMC3677446

[ece35098-bib-0081] Rovito, S. M. , Parra‐Olea, G. , Vasquez‐Almazan, C. R. , Papenfuss, T. J. , & Wake, D. B. (2009). Dramatic declines in neotropical salamander populations are an important part of the global amphibian crisis. Proceedings of the National Academy of Sciences of the United States of America, 106, 3231–3236. 10.1073/pnas.0813051106 19204286 PMC2637906

[ece35098-bib-0082] Ryan, M. J. , Lips, K. R. , & Eichholz, M. W. (2008). Decline and extirpation of an endangered Panamanian stream frog population (*Craugastor punctariolus*) due to an outbreak of chytridiomycosis. Biological Conservation, 141, 1636–1647. 10.1016/j.biocon.2008.04.014

[ece35098-bib-0083] Sala, O. E. , Chapin, F. S. , Armesto, J. J. , Berlow, E. , Bloomfield, J. , Dirzo, R. , … Wall, D. H. (2000). Global biodiversity scenarios for the year 2100. Science, 287, 1770–1774. 10.1126/science.287.5459.1770 10710299

[ece35098-bib-0084] Savage, A. E. , Zamudio, K. R. , & Sredl, M. J. (2011). Disease dynamics vary spatially and temporally in a North American amphibian. Biological Conservation, 144, 1910–1915. 10.1016/j.biocon.2011.03.018

[ece35098-bib-0085] Savage, J. M. (2002). The amphibians and reptiles of Costa Rica: A herpetofauna between two continents, between two seas. Chicago, IL: University of Chicago Press.

[ece35098-bib-0086] Scheele, B. C. , Hunter, D. A. , Brannelly, L. A. , Skerratt, L. F. , & Driscoll, D. A. (2017). Reservoir‐host amplification of disease impact in an endangered amphibian. Conservation Biology, 31, 592–600. 10.1111/cobi.12830 27594575

[ece35098-bib-0087] Scheele, B. C. , Hunter, D. A. , Grogan, L. F. , Berger, L. , Kolby, J. E. , Mcfadden, M. S. , … Driscoll, D. A. (2014). Interventions for reducing extinction risk in chytridiomycosis‐threatened amphibians. Conservation Biology, 28, 1195–1205. 10.1111/cobi.12322 24975971

[ece35098-bib-0088] Scheele, B. C. , Hunter, D. A. , Skerratt, L. F. , Brannelly, L. A. , & Driscoll, D. A. (2015). Low impact of chytridiomycosis on frog recruitment enables persistence in refuges despite high adult mortality. Biological Conservation, 182, 36–43. 10.1016/j.biocon.2014.11.032

[ece35098-bib-0089] Searle, C. L. , Biga, L. M. , Spatafora, J. W. , & Blaustein, A. R. (2011). A dilution effect in the emerging amphibian pathogen *Batrachochytrium dendrobatidis* . Proceedings of the National Academy of Sciences of the United States of America, 108, 16322–16326. 10.1073/pnas.1108490108 21930900 PMC3182747

[ece35098-bib-0090] Searle, C. L. , Gervasi, S. S. , Hua, J. , Hammond, J. I. , Relyea, R. A. , Olson, D. H. , & Blaustein, A. R. (2011). Differential host susceptibility to *Batrachochytrium dendrobatidis*, an emerging amphibian pathogen. Conservation Biology, 25, 965–974. 10.1111/j.15231739.2011.01708.x 21732979

[ece35098-bib-0091] Searle, C. L. , Mendelson, J. R. III , Green, L. E. , & Duffy, M. A. (2013). *Daphnia* predation on the amphibian chytrid fungus and its impacts on disease risk in tadpoles. Ecology and Evolution, 3, 4129–4138. 10.1002/ece3.777 24324864 PMC3853558

[ece35098-bib-0092] Skerratt, L. , Berger, L. , Hines, H. , McDonald, K. , Mendez, D. , & Speare, R. (2008). Survey protocol for detecting chytridiomycosis in all Australian frog populations. Diseases of Aquatic Organisms, 80, 85–94. 10.3354/dao01923 18717061

[ece35098-bib-0093] Skerratt, L. F. , Berger, L. , Speare, R. , Cashins, S. , McDonald, K. R. , Phillott, A. D. , … Kenyon, N. (2007). Spread of chytridiomycosis has caused the rapid global decline and extinction of frogs. EcoHealth, 4, 125–134. 10.1007/s10393-007-0093-5

[ece35098-bib-0094] Stuart, S. N. , Chanson, J. , Cox, N. A. , Young, B. E. , Rodrigues, A. S. L. , Fischman, D. , & Waller, R. (2004). Status and trends of amphibian declines and extinctions worldwide. Science, 306, 1783–1786. 10.1126/science.1103538 15486254

[ece35098-bib-0095] Thorpe, C. J. , Lewis, T. R. , Fisher, M. C. , Wierzbicki, C. J. , Kulkarni, S. , Pryce, D. , … Knight, M. E. (2018). Climate structuring of *Batrachochytrium dendrobatidis *infection in the threatened amphibians of the northern Western Ghats, India. Royal Society Open Science, 5, 180211 10.1098/rsos.180211 30110422 PMC6030269

[ece35098-bib-0096] von May, R. , Catenazzi, A. , Santa‐Cruz, R. , & Vredenburg, V. T. (2018). Microhabitat temperatures and prevalence of the pathogenic fungus *Batrachochytrium dendrobatidis* in lowland Amazonian frogs. Tropical Conservation Science, 11, 4917–13. 10.1177/1940082918797057

[ece35098-bib-0097] Voyles, J. , Young, S. , Berger, L. , Campbell, C. , Voyles, W. F. , Dinudom, A. , … Speare, R. (2009). Pathogenesis of chytridiomycosis, a cause of catastrophic amphibian declines. Science, 326, 582–585. 10.1126/science.1176765 19900897

[ece35098-bib-0098] Vredenburg, V. T. , Knapp, R. A. , Tunstall, T. S. , & Briggs, C. J. (2010). Dynamics of an emerging disease drive large‐scale amphibian population extinctions. Proceedings of the National Academy of Sciences of the United States of America, 107, 9689–9694. 10.1073/pnas.0914111107 20457913 PMC2906868

[ece35098-bib-0099] Wake, D. B. , & Vredenburg, V. T. (2008). Are we in the midst of the sixth mass extinction? A view from the world of amphibians. Proceedings of the National Academy of Sciences of the United States of America, 105, 11466–11473. 10.1073/pnas.0801921105 18695221 PMC2556420

[ece35098-bib-0100] Warne, R. W. , LaBumbard, B. , LaGrange, S. , Vredenburg, V. T. , & Catenazzi, A. (2016). Co‐infection by chytrid fungus and ranaviruses in wild and harvested frogs in the Tropical Andes. PLoS One, 11, e0145864 10.1371/journal.pone.0145864 26726999 PMC4701007

[ece35098-bib-0101] Whitfield, S. , Alvarado, G. , Abarca, J. , Zumbado‐Ulate, H. , Zuñiga, I. , Wainwright, M. , & Kerby, J. (2017). Differential patterns of *Batrachochytrium dendrobatidis* infection in relict amphibian populations following severe disease‐associated declines. Diseases of Aquatic Organisms, 126, 33–41. 10.3354/dao03154 28930083

[ece35098-bib-0102] Whitfield, S. M. , Bell, K. E. , Philippi, T. , Sasa, M. , Bolaños, F. , Chaves, G. , … Donnelly, M. A. (2007). Amphibian and reptile declines over 35 years at La Selva, Costa Rica. Proceedings of the National Academy of Sciences of the United States of America, 104, 8352–8356. 10.1073/pnas.0611256104 17449638 PMC1895953

[ece35098-bib-0103] Whitfield, S. M. , Geerdes, E. , Chacon, I. , Ballestero Rodriguez, E. , Jimenez, R. , Donnelly, M. , & Kerby, J. (2013). Infection and co‐infection by the amphibian chytrid fungus and ranavirus in wild Costa Rican frogs. Diseases of Aquatic Organisms, 104, 173–178. 10.3354/dao02598 23709470

[ece35098-bib-0104] Whitfield, S. M. , Kerby, J. , Gentry, L. R. , & Donnelly, M. A. (2012). Temporal variation in infection prevalence by the amphibian chytrid fungus in three species of frogs at La Selva, Costa Rica. Biotropica, 44, 779–784. 10.1111/j.1744-7429.2012.00872.x

[ece35098-bib-0105] Woodhams, D. C. , Bell, S. C. , Bigler, L. , Caprioli, R. M. , Chaurand, P. , Lam, B. A. , … Rollins‐Smith, L. A. (2016). Life history linked to immune investment in developing amphibians. Conservation Physiology, 4, cow025 10.1093/conphys/cow025 27928507 PMC5001151

[ece35098-bib-0106] Woodhams, D. C. , Bosch, J. , Briggs, C. J. , Cashins, S. , Davis, L. R. , Lauer, A. , … Voyles, J. (2011). Mitigating amphibian disease: Strategies to maintain wild populations and control chytridiomycosis. Frontiers in Zoology, 8, 8 10.1186/1742-9994-8-8 21496358 PMC3098159

[ece35098-bib-0107] Woodhams, D. C. , Kilburn, V. L. , Reinert, L. K. , Voyles, J. , Medina, D. , Ibánez, R. , … Rollins‐Smith, L. A. (2008). Chytridiomycosis and amphibian population declines continue to spread eastward in Panama. EcoHealth, 5, 268–274. 10.1007/s10393-008-0190-0 18807089

[ece35098-bib-0108] Young, B. E. , Lips, K. R. , Reaser, J. K. , Ibanez, R. , Salas, A. W. , Cedeño, J. R. , … Romo, D. (2001). Population declines and priorities for amphibian conservation in Latin America. Conservation Biology, 15, 1213–1223. 10.1111/j.1523-1739.2001.00218.x

[ece35098-bib-0109] Zar, J. H. (2013). *Biostatistical analysis*. Pearson Higher Ed.

[ece35098-bib-0110] Zumbado‐Ulate, H. , Bolaños, F. , Gutiérrez‐Espeleta, G. , & Puschendorf, R. (2014). Extremely low prevalence of *Batrachochytrium dendrobatidis* in frog populations from Neotropical dry forest of Costa Rica supports the existence of a climatic refuge from disease. EcoHealth, 11, 593–602. 10.1007/s10393-014-0967-2 25212725

[ece35098-bib-0111] Zumbado‐Ulate, H. , Bolaños, F. , Willink, B. , & Soley‐Guardia, F. (2011). Population status and natural history notes on the critically endangered stream‐dwelling frog *Craugastor ranoides* (Craugastoridae) in a Costa Rican tropical dry forest. Herpetological Conservation and Biology, 6, 455–464.

