# Supplementary material for: Infection with Batrachochytrium dendrobatidis is common in tropical lowland habitats: Implications for amphibian conservation
Source: Ecol Evol. 2019 Apr 1;9(8):4917–30. doi: 10.1002/ece3.5098 (PMC6476760; doi:10.1002/ece3.5098)
Supplement: Supplementary file 1 [file ECE3-9-4917-s001.docx]

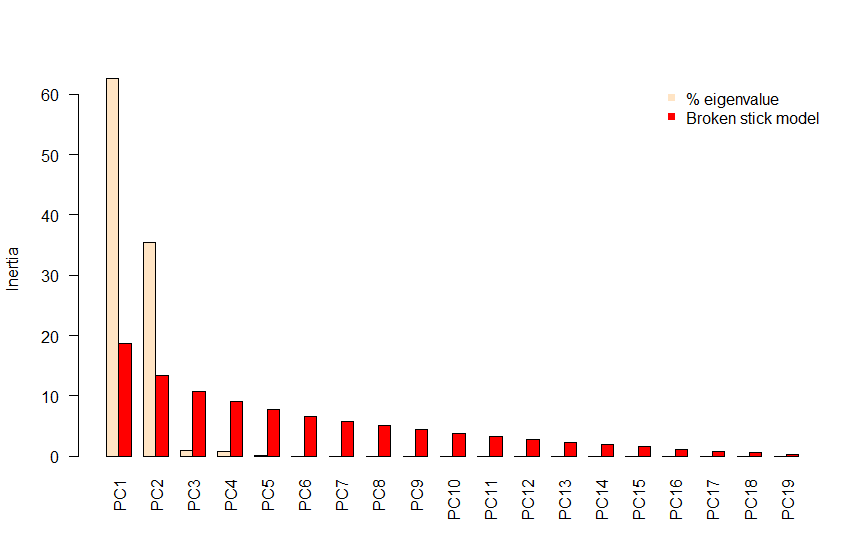


**FIGURE S1** The broken-stick plot shows that only PCA axes 1 and 2 have larger eigenvalues than the unit length (stick of unit length divided into 19 PCA axes derived from the 19 bioclimatic variables of the WorldClim dataset).


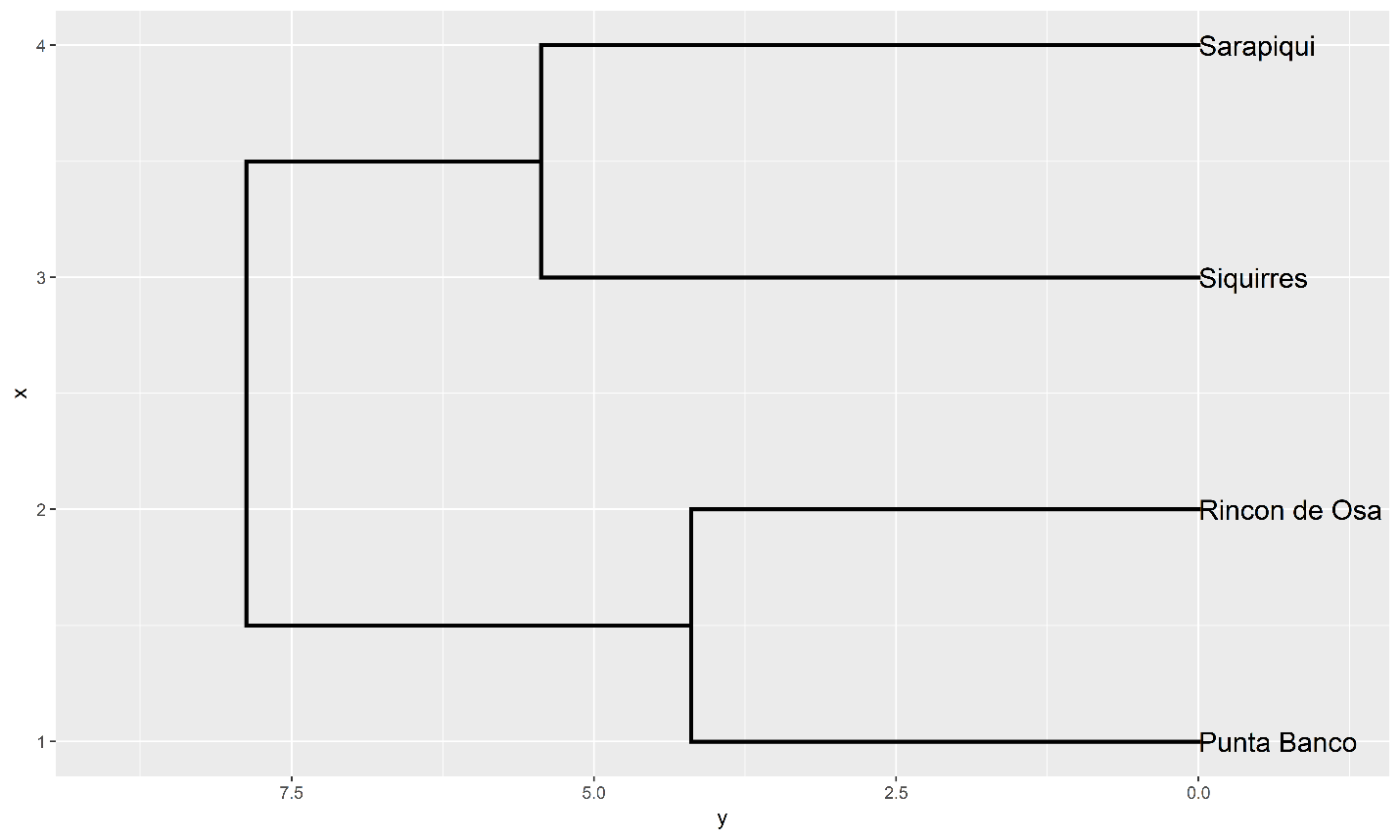


**FIGURE S2** Cluster analysis of four lowland sites in Costa Rica generated from a matrix of Euclidean distances between the centroids of climatic envelopes. Environmental values were extracted from the 19 bioclimatic variables of the WorldClim dataset. The cluster shows higher similarities between Sarapiqui and Siquirres (Caribbean side) and Rincon de Osa-Punta Banco (Pacific side).
